# The Use of HIV Pre- and Postexposure Prophylaxis Among a Web-Based Sample of HIV-Negative and Unknown Status Cisgender and Transgender Sexual Minority Men: Cross-sectional Study

**DOI:** 10.2196/31237

**Published:** 2022-12-16

**Authors:** Steven A John, K Marie Sizemore, Ruben H Jimenez, S Scott Jones, Andrew E Petroll, H Jonathon Rendina

**Affiliations:** 1 Health Intervention Sciences Group / Center for AIDS Intervention Research Department of Psychiatry and Behavioral Medicine Medical College of Wisconsin Milwaukee, WI United States; 2 Department of Psychology Hunter College of the City University of New York New York, NY United States

**Keywords:** HIV, pre-exposure prophylaxis, postexposure prophylaxis, sexual minority men, men who have sex with men

## Abstract

**Background:**

HIV disproportionately affects sexual minority men (SMM) in the United States.

**Objective:**

We sought to determine past HIV postexposure prophylaxis (PEP) use and current and prior pre-exposure prophylaxis (PrEP) use among a web-based sample of cisgender and transgender men who have sex with men.

**Methods:**

In 2019, HIV-negative and unknown status SMM (n=63,015) were recruited via geosocial networking apps, social media, and other web-based venues to participate in a brief eligibility screening survey. Individuals were asked about past PEP use and current and prior PrEP use. We examined associations of demographics, socioeconomic indicators, and recent club drug use with PEP and PrEP use, as well as the association between past PEP use and current and prior PrEP use using generalized linear models and multinomial logistic regression. Statistical significance was considered at *P*<.001, given the large sample size; 99.9% CIs are reported.

**Results:**

Prior PEP use was reported by 11.28% (7108/63,015) of the participants, with current or prior PrEP use reported by 21.95% (13,832/63,015) and 8.12% (5118/63,015), respectively. Nearly half (3268/7108, 46%) of the past PEP users were current PrEP users, and another 39.9% (2836/7108) of the participants who reported past PEP use also reported prior PrEP use. In multivariable analysis, past PEP use was associated with current (relative risk ratio [RRR] 23.53, 99.9% CI 14.03-39.46) and prior PrEP use (RRR 52.14, 99.9% CI 29.39-92.50). Compared with White men, Black men had higher prevalence of past PEP use and current PrEP use, Latino men had higher prevalence of PEP use but no significant difference in PrEP use, and those identifying as another race or ethnicity reported higher prevalence of past PEP use and lower current PrEP use. Past PEP use and current PrEP use were highest in the Northeast, with participants in the Midwest and South reporting significantly lower PEP and PrEP use. A significant interaction of Black race by past PEP use with current PrEP use was found (RRR 0.57, 99.9% CI 0.37-0.87), indicating that Black men who previously used PEP were less likely to report current PrEP use. Participants who reported recent club drug use were significantly more likely to report past PEP use and current or prior PrEP use than those without recent club drug use.

**Conclusions:**

PrEP use continues to be the predominant HIV prevention strategy for SMM compared with PEP use. Higher rates of past PEP use and current PrEP use among Black SMM are noteworthy, given the disproportionate burden of HIV. Nonetheless, understanding why Black men who previously used PEP are less likely to report current PrEP use is an important avenue for future research.

## Introduction

### Background

HIV disproportionately affects cisgender and transgender men who have sex with men—referred to herein as sexual minority men (SMM)—in the United States [1,2]. Despite decreasing HIV incidence nationally, cisgender SMM accounted for 68% of the sexually transmitted HIV incidence in 2019 [1]. HIV prevalence is estimated at 3.2% among transgender men, and 55.2% of the HIV-negative or unknown status transgender SMM could benefit from biomedical HIV prevention [3]. In 2019, the *Ending the HIV Epidemic: A Plan for America* was announced, with priorities that include expanding biomedical HIV prevention [4]. HIV pre-exposure prophylaxis (PrEP) is a method of biomedical HIV prevention that includes taking antiretrovirals once daily—as approved by the US Food and Drug Administration [5] with supporting guidelines from the Centers for Disease Control and Prevention [6]—or alternative dosing strategies (eg, 2-1-1) found to be highly effective in preventing HIV [7-10]. Nonetheless, engagement in anal sex can be unanticipated—both consensual and nonconsensual—and alternative options are needed after such encounters with an HIV-positive or unknown status partner.

Nonoccupational HIV postexposure prophylaxis (PEP) is a highly effective method of secondary HIV prevention [11-17] and can be administered within 72 hours after exposure or potential exposure to HIV. PEP, a 28-day strategy that includes taking a 3- or 4-drug regimen of HIV antiretrovirals after exposure [18,19], has been recommended as a strategy for HIV prevention by the Centers for Disease Control and Prevention since 2005 [20]. By contrast, PrEP is a 2-drug combination using emtricitabine with either tenofovir disoproxil fumarate or tenofovir alafenamide [21]. When indicated, individuals who complete the 28-day PEP regimen are then recommended to transition immediately to PrEP [6,22]. Strategies to support successful PEP-to-PrEP transition are beginning to be implemented in clinical settings, with initial data indicating high success [23].

### Study Hypothesis

Many individuals who use PEP after potential sexual exposure to HIV are appropriate candidates to initiate PrEP upon completion of PEP [6,22], but research is limited on PEP uptake among SMM. As such, we sought to determine lifetime use of PEP among a large nationwide sample of SMM recruited on the internet. We hypothesized that SMM who had prior experience with PEP would report higher rates of PrEP use than SMM who had not used PEP. Given the dearth of data on PEP use among SMM, we also explored prior PEP use by demographic characteristics, health insurance status, socioeconomic status, and club drug use; in addition, we examined the effect of these factors on PrEP uptake.

## Methods

### Participants and Procedures

Participants were recruited via geosocial networking apps, social media, and other web-based venues targeting SMM between January 1, 2019, and December 31, 2019, to participate in a brief (5-10 minutes) screening survey used to determine eligibility for multiple paid research studies. Only individuals aged ≥13 years were eligible to take the screening survey. To be eligible for this analysis, participants were required to (1) identify as male (inclusive of transgender men), (2) report a male sexual partner in the past 6 months or a main partner who identified as male, (3) self-report HIV-negative or unknown status, and (4) reside in the United States, including Puerto Rico and other territories. On the basis of the recruitment procedures, advertisements, and venues targeted to men, women (inclusive of transgender women) were excluded from the analysis. Cisgender and transgender SMM were the focus of our analysis, given the disproportionate burden of HIV incidence in the United States [1,2]. All adolescent SMM were included in this analysis; children and adolescents are included in current PEP guidelines based on supporting safety data collected among young people [18], and PrEP is approved for use among minors weighing ≥35 kg [24]. Fraudulent responses were minimized by excluding any information of eligibility criteria in study advertisements and referral mechanisms and offering no incentive for completion of this screening survey. Potential duplicate responses were identified by corresponding birth month and year, zip code, HIV status, race, and ethnicity. Flagged cases were further screened by examining other demographic variables and metadata (eg, device and browser information) before being considered for removal, as recommended previously [25].

### Ethical Considerations

An alteration of informed consent and assent was approved for this study, wherein participants agreed to participate after reading an informational letter describing the study procedures, risks, and benefits; parental permission was waived for all minors. No incentive was provided for participation in this screening survey. Surveys were conducted using Qualtrics, which provides Health Insurance Portability and Accountability Act privacy protection standards, and contact information was collected separately from survey data to reduce the risk harm in the case of loss of confidentiality. All study procedures were approved by the institutional review board of the City University of New York (319487).

### Measures

Participants were asked to report their age, sexual orientation, gender, race and ethnicity, health insurance status, and location of residence in the United States. Age was categorized for analysis using thresholds used in the US HIV Surveillance Report [1]. Gender was determined using a 2-step approach: participants indicated their sex assigned at birth with male and female response categories, and current gender identity was indicated by their response to the question “What is your current gender identity?” The response categories were male, female, and transgender. We regret the exclusion of additional gender identities in our response options, including but not limited to genderqueer, nonbinary, and 2 spirit. Individuals who reported being assigned female sex at birth and currently identified as male or transgender were coded as transgender men. Individuals were asked to indicate their race and ethnicity, and participants in the multiracial category either indicated >1 race or selected a multiracial category. Participants were also asked about their perceived socioeconomic status using the MacArthur Scale of Subjective Social Status [26], which measures participants’ perceived socioeconomic rank compared with others, with 1 being the lowest and 10 being the highest. Individuals were coded as having used club drugs if they reported using any of the following substances in the past 90 days: crack and cocaine; 3,4-methylenedioxy-methamphetamine (MMDA); gamma-hydroxybutyrate (GHB); ketamine; or methamphetamine [27]. Participants were provided a brief introduction regarding PrEP and asked the following question about PrEP use: “Have you ever been prescribed HIV medications (e.g., Truvada) for use as PrEP (pre-exposure prophylaxis)?” The response options included (1) *Yes, I am currently on PrEP*; (2) *Yes, but I am no longer taking PrEP*; and (3) *No, I’ve never taken PrEP* [28,29]. Similarly, participants were provided a brief introduction regarding PEP and asked the following question: “Have you ever been prescribed PEP?” The response options included (1) *Yes, within the past 6 months*; (2) *Yes, more than 6 months ago*; and (3) *No, never*. PEP use was coded into past lifetime use (*yes* or *no*).

### Data Analysis

Descriptive statistics were reported using frequency measures. For the past PEP use outcome, bivariate analyses were conducted using generalized linear models with log link function and Poisson distribution to produce prevalence ratios. We then examined associations between demographics and club drug use on ever using PEP using fully adjusted generalized linear models with log link function and Poisson distribution. For the current and prior PrEP use outcomes, bivariate analyses were conducted using multinomial logistic regression, which produced relative risk ratios (RRRs). We then examined associations among demographics, club drug use, and past PEP use with current and prior PrEP use using fully adjusted multinomial logistic regression; *never used PrEP* was the referent in the past PrEP use multinomial model. We removed insurance status and the socioeconomic status score from all adjusted models to reduce overadjustment bias [30] because of their role as hypothesized intermediate variables in the causal pathways between race and ethnicity (via racism) and PEP or PrEP use; insurance status and socioeconomic status score were thus removed to improve theoretical model precision. Interactions between race and ethnicity and past PEP use with PrEP use were explored by adding two interaction terms to the PrEP models: (1) Black, non-Hispanic×past PEP use and (2) Latino or Hispanic×past PEP use. Statistical significance was tested at α=.001 because of the large sample size, and unadjusted and adjusted prevalence ratios are reported with 99.9% CIs.

## Results

### Participant Characteristics

Recruitment activities resulted in 160,581 unique link clicks, with 120,274 (74.9%) participants agreeing to participate in the survey. Among those who agreed, 76.1% (91,526/120,274) completed the survey or provided data sufficient for analysis. Of these, 3.87% (3538/91,526) were ineligible by gender, 10.94% (10,011/91,526) did not report a recent male sexual partner or a main partner who identified as male, and 19.91% (18,219/91,526) self-reported living with HIV; individuals could be considered ineligible by ≥1 of the criteria. Thus, of the 91,526 SMM who agreed to participate and provided data sufficient for analysis, 63,015 (68.85%) were eligible for this analysis. The average age of respondents was 33.1 (SD 12.0) years (median 30, range 13-80; Table 1). Most of the participants identified as gay (45,251/63,015, 71.81%) or bisexual (15,129/63,015, 24%), and nearly all (62,446/63,015, 99.1%) identified as cisgender men. Past PEP use was reported by 11.28% (7108/63,015) of the participants, and 21.95% (13,832/63,015) and 8.12% (5118/63,015) reported current and prior PrEP use, respectively. Nearly half (3268/7108, 46%) of the past PEP users were current PrEP users, and another 39.9% (2836/7108) of the participants who reported past PEP use also reported prior PrEP use. Refer to Tables 1 and 2 for full sample characteristics.

In bivariate analyses, significant differences in past PEP use prevalence were found by age, sexual orientation, US region, race and ethnicity, health insurance status, and recent club drug use (Table 1). In addition, significant differences in PrEP uptake were found by age, sexual orientation, gender, US region, race and ethnicity, health insurance status, recent club drug use, and past PEP use (Table 2). Socioeconomic status was significant in both models, but effect sizes did not indicate a meaningful effect (Tables 1 and 2).

**Table 1:** Demographics, socioeconomic status indicators, club drug use, and current use of pre-exposure prophylaxis (PrEP) and their bivariate associations with previous postexposure prophylaxis (PEP) use (N=63,015).

				Values		Past PEP use				
						Values (n=7108)		Prevalence ratio (99.9% CI)		*P* value
**Categorical variables, n (%)^a^**										
	**Age (years; mean 33.12, SD 11.86; median 30, range 13-80)**									
		13 to 24	16,641 (26.41)		1160 (16.32)		N/A^b^		N/A	
		25 to 34	23,432 (37.18)		3200 (45.02)		*1.96*^c^ (1.76-2.18)		<.001	
		35 to 44	11,502 (18.25)		1609 (22.64)		*2.01* (1.78-2.26)		<.001	
		≥45	11,440 (18.15)		1139 (16.05)		*1.43* (1.25-1.63)		<.001	
	**Sexual orientation identity**									
		Gay	45,251 (71.81)		5875 (82.65)		N/A		N/A	
		Bisexual	15,129 (24)		929 (13.07)		*0.47* (0.42-0.53)		<.001	
		Queer	1758 (2.79)		266 (3.74)		*1.17* (0.96-1.41)		.008	
		Straight	877 (1.39)		38 (0.53)		*0.33* (0.20-0.56)		<.001	
	**Gender**									
		Cisgender man	62,446 (99.1)		7041 (99.06)		N/A		N/A	
		Transgender man	569 (0.9)		67 (0.94)		1.04 (0.72-1.53)		.71	
	**Region**									
		Northeast	12,823 (20.35)		1867 (26.27)		N/A		N/A	
		Midwest	11,359 (18.03)		1071 (15.07)		*0.65* (0.58-0.73)		<.001	
		South	21,087 (33.46)		1936 (27.24)		*0.63* (0.57-0.70)		<.001	
		West	17,418 (27.64)		2209 (31.08)		*0.87* (0.79-0.96)		<.001	
		US possession	255 (0.4)		16 (0.23)		*0.43* (0.19-0.96)		<.001	
		Military overseas	29 (0.05)		3 (0.04)		0.71 (0.12-4.30)		.53	
		Unknown	44 (0.07)		6 (0.08)		0.94 (0.27-3.27)		.86	
	**Race and ethnicity**									
		Black, non-Hispanic	6628 (10.52)		774 (10.89)		*1.17* (1.04-1.33)		<.001	
		Latino or Hispanic	11,092 (17.6)		1474 (20.74)		*1.34* (1.21-1.47)		<.001	
		Multiracial	6385 (10.13)		909 (12.79)		0.61 (0.23-1.58)		.09	
		White, non-Hispanic	35,046 (55.61)		3485 (49.03)		N/A		N/A	
		Another	3864 (6.13)		466 (6.56)		*1.36* (1.23-1.50)		<.001	
	**Health insurance status**									
		Has private health insurance	39,071 (62)		4352 (61.23)		*1.12* (1.01-1.23)		<.001	
		Has public health insurance (eg, Medicaid)	11,151 (17.7)		1481 (20.84)		*1.33* (1.18-1.50)		<.001	
		Uninsured	12,793 (20.3)		1257 (17.68)		N/A		N/A	
	**Any club drug use (past 90 days)^d^**									
		No	50,411 (80)		5222 (73.47)		N/A		N/A	
		Yes	12,604 (20)		1886 (26.53)		*1.45* (1.33-1.57)		<.001	
	**PrEP** **use status**									
		Never used	44,065 (69.93)		1003 (14.11)		N/A		N/A	
		Prior use	5118 (8.12)		2837 (39.91)		*24.35* (21.80-27.20)		<.001	
		Current use	13,832 (21.95)		3268 (45.98)		*10.38* (9.26-11.64)		<.001	
**Continuous variable, mean (SD)**										
	Socioeconomic status ladder (range 1-10)		6.66 (25.33)		7.69 (38.93)		*1.00* (1.00-1.00)		<.001	

^a^Percentages may not add up to 100 because of rounding.

^b^N/A: not applicable.

^c^Italicized prevalence ratio values are significant at *P*<.001.

^d^Club drugs include crack and cocaine; 3,4-methylenedioxy-methamphetamine (MMDA); gamma-hydroxybutyrate (GHB); ketamine; and methamphetamine.

**Table 2:** Demographics, socioeconomic status indicators, club drug use, and past postexposure prophylaxis (PEP) use and their bivariate associations with current and prior pre-exposure prophylaxis (PrEP) use compared with never used PrEP (N=63,015).

			Current PrEP use				Prior PrEP use				
			Values (n=13,832)	RRR^a^ (99.9% CI)	*P* value	Values (n=5118)		RRR (99.9% CI)		*P* value	
**Categorical variables, n (%)^b^**											
	**Age (years)**										
		13 to 24	1911 (13.82)	N/A^c^	N/A	959 (18.74)		N/A		N/A	
		25 to 34	5807 (41.98)	*2.75*^d^ (2.50-3.03)	<.001	2414 (47.17)		*2.28* (2.00-2.60)		<.001	
		35 to 44	3273 (23.66)	*3.28* (2.95-3.65)	<.001	1044 (20.4)		*2.09* (1.79-2.44)		<.001	
		≥45	2841 (20.54)	*2.59* (2.33-2.89)	<.001	701 (13.7)		*1.23* (1.08-1.51)		<.001	
	**Sexual orientation identity**										
		Gay	11,792 (85.25)	N/A	N/A	4178 (81.63)		N/A		N/A	
		Bisexual	1533 (11.08)	*0.30* (0.27-0.33)	<.001	711 (13.89)			*0.39* (0.34-0.44)		<.001
		Queer	479 (3.46)	1.11 (0.93-1.34)	.06	211 (4.12)			1.39 (1.07-1.79)		<.001
		Straight	28 (0.2)	*0.08* (0.04-0.16)	<.001	18 (0.35)			*0.15* (0.07-0.33)		<.001
	**Gender**										
		Cisgender man	13,748 (99.39)	N/A	N/A	5069 (99.04)			N/A		N/A
		Transgender man	84 (0.61)	*0.61* (0.41-0.91)	<.001	49 (0.96)			0.97 (0.59-1.59)		.83
	**Region**										
		Northeast	3294 (23.81)	N/A	N/A	1562 (30.52)			N/A		N/A
		Midwest	2307 (16.68)	*0.71* (0.64-0.78)	<.001	1240 (24.23)			*0.68* (0.58-0.79)		<.001
		South	4054 (29.31)	*0.66* (0.60-0.72)	<.001	833 (16.28)			*0.63* (0.55-0.72)		<.001
		West	4131 (29.87)	*0.89* (0.81-0.97)	<.001	1466 (28.64)			0.89 (0.78-1.02)		.004
		US possession	35 (0.25)	*0.42* (0.23-0.77)	<.001	10 (0.2)			*0.32* (0.11-0.93)		<.001
		Military overseas	6 (0.04)	0.76 (0.16-3.50)	.55	3 (0.06)			1.00 (0.13-7.71)		.99
		Unknown	5 (0.04)	0.36 (0.07-1.74)	.03	4 (0.08)			0.76 (0.13-4.35)		.61
	**Race and ethnicity**										
		Black, non-Hispanic	1311 (9.48)	*0.84* (0.75-0.94)	<.001	540 (8.1)			1.07 (0.90-1.26)		.21
		Latino or Hispanic	2359 (17.05)	0.94 (0.86-1.03)	.02	1050 (9.5)			*1.29* (1.13-1.46)		<.001
		Multiracial	1329 (9.61)	*0.21* (0.06-0.71)	<.001	630 (9.9)			0.25 (0.05-1.33)		.006
		White, non-Hispanic	7996 (57.81)	N/A	N/A	2596 (7.4)			N/A		N/A
		Another	837 (6.05)	0.94 (0.86-1.03)	.02	302 (7.8)			*1.24* (1.09-1.42)		<.001
	**Health insurance status**										
		Has private health insurance	10,118 (73.15)	*2.79* (2.52-3.08)	<.001	2932 (7.5)			0.96 (0.85-1.08)		.28
		Has public health insurance (eg, Medicaid)	2293 (16.58)	*2.09* (1.85-2.36)	<.001	993 (8.9)			1.08 (0.93-1.25)		.10
		Uninsured	1421 (10.27)	N/A	N/A	1193 (9.3)			N/A		N/A
	**Any club drug use (past 90 days)^e^**										
		No	10,512 (76)	N/A	N/A	3719 (7.4)			N/A		N/A
		Yes	3320 (24)	*1.45* (1.34-1.57)	<.001	1399 (11.1)			*1.73* (1.55-1.93)		<.001
	**Past** **PEP** **use**										
		No	10,564 (76.37)	N/A	N/A	2281 (4.1)			N/A		N/A
		Yes	3268 (23.63)	*13.28* (11.73-15.04)	<.001	2837 (39.9)			*53.40* (46.42-61.43)		<.001
**Continuous variable, mean (SD)**											
	Socioeconomic status ladder (range 1-10)		7.47 (32.65)	*1.001* (1.000-1.003)	<.001	6.90 (31.02)			*1.001* (0.999-1.003)		.09

^a^RRR: relative risk ratio.

^b^Percentages may not add up to 100 because of rounding.

^c^N/A: not applicable.

^d^Italicized relative risk ratio values are significant at *P*<.001.

^e^Club drugs include crack and cocaine; 3,4-methylenedioxy-methamphetamine (MMDA); gamma-hydroxybutyrate (GHB); ketamine; and methamphetamine.

### Multivariable Analyses

In multivariable analyses (Tables 2 and 3), past PEP use was associated with current (RRR 23.53, 99.9% CI 14.03-39.46) and prior PrEP use (RRR 52.14, 99.9% CI 29.39-92.50). Compared with White men, Black men had higher prevalence of past PEP use and current PrEP use, Latino men had higher prevalence of PEP use but no significant difference in PrEP use, and those identifying as another race or ethnicity reported higher prevalence of past PEP use and lower current PrEP use. Compared with White men, multiracial men had no significant difference in PEP or PrEP use. Past PEP use and current PrEP use were highest in the Northeast, with participants in the Midwest and South reporting significantly lower PEP and PrEP use. Men living in the West had significantly lower prevalence of past PEP use compared with men in the Northeast, but no significant difference in PrEP use was observed. Individuals living in a US possession also had significantly lower prevalence of past PEP use, as well as lower likelihood of current PrEP use. A significant interaction of Black race by past PEP use with current PrEP use was found (RRR 0.57, 99.9% CI 0.37-0.87), indicating that Black men who previously used PEP were less likely to report current PrEP use. Participants who reported recent club drug use were significantly more likely to report past PEP use and current or prior PrEP use than those without recent use. Refer to Tables 2 and 3 for full multivariable results.

**Table 3:** Results from generalized linear models with log link function and Poisson distribution predicting past postexposure prophylaxis (PEP) use and multinomial logistic regression comparing current and prior pre-exposure prophylaxis (PrEP) use with never used PrEP (N=63,015).

Categorical variables			Past PEP use (referent: never used)				PrEP use (referent: never used)								
			PR^a^ (99.9% CI)			*P* value	Current use						Prior use		
							RRR^b^ (99.9% CI)			*P* value			RRR (99.9% CI)	*P* value	
**Age (years)**															
	13 to 24		N/A^c^			N/A	N/A			N/A			N/A	N/A	
	25 to 34		*1.96*^d^ (1.74-2.22)			<.001	*2.43* (2.20-2.69)			<.001			*1.23* (1.02-1.50)	<.001	
	35 to 44		*2.09* (1.83-2.40)			<.001	*2.91* (2.60-3.26)			<.001			*1.72* (1.44-2.06)	<.001	
	≥45		*1.59* (1.37-1.85)			<.001	*2.46* (2.19-2.76)			<.001			*1.88* (1.61-2.18)	<.001	
**Sexual orientation identity**															
	Gay			N/A	N/A		N/A		N/A		N/A			N/A	
	Bisexual			*0.46* (0.41-0.52)	<.001		*0.32* (0.29-3.36)		<.001		*0.45* (0.38-0.52)			<.001	
	Queer			1.13 (0.89-1.43)	.10		*1.22* (0.99-1.49)		.001		1.34 (0.99-1.82)			.001	
	Straight			*0.30* (0.17-0.52)	<.001		*0.08* (0.04-0.16)		<.001		*0.15* (0.06-0.35)			<.001	
**Gender**															
	Cisgender man		N/A			N/A	N/A			N/A		N/A			N/A
	Transgender man		1.19 (0.75-1.89)			.21	0.71 (0.45-1.10)			.009		0.85 (0.46-1.57)			.39
**Region**															
		Northeast	N/A			N/A		N/A		N/A		N/A			N/A
		Midwest	*0.66* (0.58-0.76)			<.001		*0.78* (0.70-0.88)		<.001		0.85 (0.71-1.01)			.002
		South	*0.61* (0.54-0.69)			<.001		*0.72* (0.66-0.79)		<.001		*0.77* (0.66-0.90)			<.001
	West		*0.81* (0.72-0.91)			<.001		0.92 (0.83-1.02)		.006		0.93 (0.80-1.09)			.14
	US possession		*0.35* (0.15-0.82)			<.001		*0.45* (0.24-0.86)		<.001		0.38 (0.12-1.23)			.007
	Military overseas		0.70 (0.09-5.21)			.56		0.91 (0.18-4.72)		.85		1.36 (0.13-14.60)			.67
	Unknown		1.14 (0.25-5.09)			.78		0.40 (0.07-2.18)		.07		0.75 (0.09-6.12)			.65
**Race and ethnicity**															
	Black, non-Hispanic		*1.36* (1.18-1.57)			<.001		*1.60* (1.06-2.42)		<.001		1.29 (0.85-1.97)			.002
	Latino or Hispanic		*1.41* (1.26-1.58)			<.001		1.08 (0.79-1.48)		.41		1.07 (0.78-1.47)			.49
	Multiracial		1.10 (0.37-3.21)			.78		0.47 (0.14-1.64)		.47		0.37 (0.06-2.28)			.07
	White, non-Hispanic		N/A			N/A		N/A		N/A		N/A			N/A
	Another		*1.38* (1.23-1.56)			<.001		*0.90* (0.81-1.00)		<.001		0.98 (0.83-1.14)			.60
**Any club drug use (past 90 days)^e^**															
	No		N/A			N/A		N/A		N/A		N/A			N/A
	Yes		*1.40* (1.27-1.55)			<.001		*1.29* (1.18-1.40)		<.001		*1.47* (1.29-1.67)			<.001
**Past** **PEP** **use**															
	No		N/A			N/A		N/A		N/A		N/A			N/A
	Yes		N/A			N/A		*23.53* (14.03-39.46)		<.001		*52.14* (29.39-92.50)			<.001
Black, non-Hispanic×past PEP use			N/A			N/A		*0.57* (0.37-0.87)		<.001		0.87 (0.54-1.40)			.34
Latino or Hispanic×past PEP use			N/A			N/A		0.84 (0.61-1.16)		.07		1.09 (0.76-1.56)			.44

^a^PR*:* prevalence ratio.

^b^RRR*:* relative risk ratio.

^c^N/A: not applicable.

^d^Italicized prevalence ratio and relative risk ratio values are significant at *P*<.001.

^e^Club drugs include crack and cocaine; 3,4-methylenedioxy-methamphetamine (MMDA); gamma-hydroxybutyrate (GHB); ketamine; and methamphetamine.

## Discussion

### Principal Findings

We sought to determine lifetime use of PEP among SMM and hypothesized that SMM who had prior experience with PEP would report higher rates of PrEP use than SMM who had not used PEP. Specifically, we found that 11.28% (7108/63,015) of the participants reported past PEP use, but PrEP use was the more commonly used method of HIV prevention. As hypothesized, we found that men with a history of PEP use were more likely to report current PrEP use. When considering both current and prior PrEP use, 85.9% (6106/7108) of the past PEP users had also used PrEP currently or previously. As such, PEP use could be a gateway to PrEP use as a PEP-to-PrEP pathway to biomedical HIV prevention, supported by current guidelines and recommendations [6,22] as well as current PrEP and PEP implementation strategies [23].

We find it plausible that individuals who have previous experience taking antiretrovirals for PEP could have fewer barriers to taking PrEP. SMM frequently cite concerns about potential side effects with taking PrEP [31,32], concerns that could potentially diminish after the experience of taking PEP. Moreover, PEP is frequently obtained in urgent scenarios, given the short time interval to initiation, offering a cue to action for ongoing HIV prevention. PEP users are also most often put in contact with providers who could become their prescribers of PrEP. Further research is needed to explore the PEP-to-PrEP pathway to biomedical HIV prevention, including reasons for uptake of, or declining, PrEP, but our findings illustrate that nearly half (3268/7108, 46%) of the past PEP users are currently taking PrEP. Moreover, our findings about lifetime PEP uptake are higher than prior reports of PEP use more broadly, where a pooled estimate of PEP use was 5.8% in high-income countries in a systematic review [33]; yet, our nationwide findings find concordance with increasing uptake over time, including similar rates of PEP use reported among young SMM (ie, 11.5% [34]) and young SMM of color (ie, 15.3% [35]) in New York City—a high-resource area for HIV prevention.

Although our cross-sectional analysis is limited in our ability to distinguish temporality between past PEP use and prior PrEP use, our findings illustrate the potential need for further research in this area. Individuals who had previously used PEP had a >50-fold likelihood of prior PrEP use. Further research is needed to identify how PEP and PrEP can be used interchangeably to support individuals’ HIV prevention goals. Specifically, PrEP use is intended to be flexible based on potential vulnerability to HIV infection, where individuals can discontinue daily PrEP during breaks in sexual behavior or in combination with other HIV prevention strategies, including mutual monogamy with a recently tested HIV-negative partner or a partner with an undetectable viral load (ie, HIV positive with sustained viral suppression). Research is robust on reasons for discontinuing PrEP use, such as lower perceived risk and challenges with cost and access [36-40]. Moreover, gaps in PrEP use are normalized and encouraged when biomedical HIV prevention is not necessary because many individuals report changes in sexual behavior and perceived HIV risk over time [41-43]. Advancements in 2-1-1 PrEP dosing also present new opportunities where unanticipated sexual behavior may result in condomless anal sex without PrEP protection—necessitating the potential need for PEP before PrEP reinitiation. Thus, PEP adds to the HIV prevention toolbox in combination with PrEP, but a study of how PEP is used among individuals who discontinued PrEP is needed.

PEP seems to potentially have a small role in combating disparities in HIV incidence, where Black SMM, Latino SMM, and SMM identifying as another race or ethnicity reported higher prevalence of past PEP use than White SMM. Disparities in PrEP uptake are well documented, with fewer Black and Latino SMM using PrEP compared with White SMM [44,45], despite accounting for 37% and 21% of HIV incidence among gay and bisexual men, respectively [46]. In crude statistics, our findings also indicate that fewer Black and Latino SMM are using PrEP compared with White SMM; yet, the magnitude of this difference is smaller within this web-based sample than within the aggregated commercial pharmacy data reported by AIDSVu [47]. HIV incidence decreased 15% among White SMM between 2014 and 2018, but HIV incidence remained stable for Black and Latino SMM [46], likely resulting from inequitable access and barriers to HIV treatment and PrEP. PEP is unique in its use because it can be dispensed in a single prescription, including all pills for the 28-day regimen, avoiding some of the barriers to PrEP uptake and persistence that include quarterly visits to a provider and ongoing navigation of insurance and copay assistance programs [39,48]. As such, PEP is especially important as a mechanism of HIV prevention because of notable gaps in, and barriers to, PrEP use among SMM.

PEP users in our web-based sample of SMM had a similar profile, by age and sexual orientation, as PrEP users as also reported in other samples. We found SMM aged <25 years to have lower prevalence of past PEP use than older SMM aged 25 to 44 years, similar to disparities in PrEP uptake and persistence [45,49]; yet, this is expected in lifetime use statistics, given that older people have had more time to access these interventions, especially as the length of time that PEP and PrEP have been available is increasing. Nonetheless, specific barriers to PrEP use among young SMM include privacy and insurance issues, including the challenges of living with parents and being on the parents’ insurance plan, high cost of PrEP, and perceived adherence challenges [50-52]. Moreover, we found that those who identified as bisexual had lower prevalence of past PEP use than those who identified as gay, aligning with disparities in PrEP uptake where bisexual men were less likely to take PrEP than their gay counterparts in other research [45]. PrEP stigma is pervasive and a known barrier to PrEP uptake [53], compounded with homonegativity and the enduring effects of early advertising of PrEP specifically targeted to men who have sex with men [53,54]. As such, structural interventions are needed to make PEP more accessible to younger SMM and to prioritize bisexual SMM in HIV prevention efforts, given the suboptimal biomedical HIV prevention uptake to date.

Our large sample provided an opportunity to compare PEP and PrEP uptake between cisgender and transgender SMM. Specifically, we found no difference in past PEP use between cisgender and transgender SMM, but fewer transgender SMM reported current PrEP use compared with cisgender SMM by a large magnitude (84/569, 14.8%, vs 13,748/62,446, 22%, respectively). Prior research found that nearly two-thirds of transgender men who have sex with men met clinical guidelines for PrEP in 2017; yet, uptake was reported by only 21.8% of transgender SMM [55]. Our findings here from 2019 found lower rates of both current and prior PrEP use among transgender SMM perhaps because of sampling strategies. We focused exclusively on web-based recruitment, whereas Reisner et al [55] also recruited via social networks, engagement with community-based organizations, and outreach at a Philadelphia-based transgender health-focused conference. Similarly, 26.1% of the transgender men recruited on the internet from October 2017 to May 2018 ever reported PrEP use [40]; yet, these findings were not disaggregated by current or prior PrEP use and are similar to our study’s 23.4% (133/569) who reported ever being prescribed PrEP. Further efforts are needed to target barriers to PrEP uptake, such as reducing potential misconceptions about interactions with gender-affirming therapy, establishing trusting relationships between medical institutions and transgender patients, and reducing PrEP stigma negatively affecting PrEP knowledge and attitudes as thematically organized by a systematic review of the literature [56].

Finally, we found that SMM who had recently engaged in club drug use were more likely to report past PEP use and current or prior PrEP use in concordance with prior research [57]. There is substantial evidence that club drug use, including the use of methamphetamine and other stimulants, is strongly associated with condomless anal sex as well as HIV and sexually transmitted infection acquisition among SMM [27,57-60]. Moreover, researchers have identified altered rectal cytokines among SMM who used stimulants [61]. Researchers suggest the confluence of condomless anal sex and dysregulated rectal immune functioning as an important potential driver of HIV transmission among SMM who use stimulants [62]. As such, SMM who use club drugs are a priority population for biomedical HIV prevention. Our findings regarding greater engagement in biomedical HIV prevention among club drug users is promising because current PEP and PrEP implementation efforts are reaching SMM at heightened vulnerability to HIV via substance use. Importantly, our findings align with previous reports about PEP use among young SMM in New York City, where researchers found that young SMM who used methamphetamine had >6 times higher odds of past PEP use.

### Limitations

Our research is not without limitations. First, we recruited a convenience sample on the internet without incentivizing participation, which may have resulted in biased enrollment and introduced selection bias, potentially limiting the generalizability of the findings. Second, there is a potential for recall bias, especially related to lifetime past PEP use. Third, social desirability bias cannot be ruled out, which may have resulted in, for example, higher endorsement of PEP and PrEP use and lower reports of substance use. Finally, we conducted a cross-sectional analysis describing PEP and PrEP use with potential issues related to temporality, especially regarding past PEP and prior PrEP use. Additional longitudinal and qualitative research is needed to better understand PEP use and its potential impact on PrEP uptake or discontinuation.

### Conclusions

PrEP use was the predominate HIV prevention strategy reported in our web-based sample of SMM compared with PEP; yet, our findings indicate that PEP use could be a gateway to PrEP use because nearly half (3268/7108, 46%) of the current PrEP users reported prior use of PEP. Advertising and prescribing PEP could also support efforts to increase PrEP uptake and sustain HIV prevention during breaks or interruptions in daily or intermittent PrEP use. Further research is needed to better understand and support this phenomenon to maximize the use of currently available biomedical HIV prevention tools.

## Abbreviations

GHB: gamma-hydroxybutyrate
MMDA: 3,4-methylenedioxy-methamphetamine
PEP: postexposure prophylaxis
PrEP: pre-exposure prophylaxis
RRR: relative risk ratio
SMM: sexual minority men
